# Bi-atrial function and its relation with biventricular function and clinical parameters in patients operated for tetralogy of Fallot

**DOI:** 10.1186/1532-429X-14-S1-P124

**Published:** 2012-02-01

**Authors:** Saskia E Luijnenburg, Rosanne Peters, Rob J van der Geest, Adriaan Moelker, Jolien W Roos-Hesselink, Yolanda B de Rijke, Hubert W Vliegen, Barbara J Mulder, Willem A Helbing

**Affiliations:** 1 Pediatric Cardiology, Erasmus Medical Center - Sophia Children's Hospital, Rotterdam, Netherlands; 2 Radiology, Erasmus Medical Center, Rotterdam, Netherlands; 3 Radiology, Leiden University Medical Center, Leiden, Netherlands; 4 Cardiology, Erasmus Medical Center, Rotterdam, Netherlands; 5 Clinical Chemistry, Erasmus Medical Center, Rotterdam, Netherlands; 6 Cardiology, Leiden University Medical Center, Leiden, Netherlands; 7 Cardiology, Academic Medical Center, Amsterdam, Netherlands

## Background

Biventricular size and function have been studied extensively in patients after tetralogy of Fallot (TOF) repair, but little is known about atrial size and function. The atria play a crucial role in ventricular filling during diastole, and abnormalities in atrial size and function may reflect ventricular diastolic dysfunction. Diastolic dysfunction may precede systolic dysfunction and may therefore play an important role in the early detection of ventricular dysfunction.

### Aim

We assessed bi-atrial size and function in patients after TOF repair, and evaluated relationships with biventricular systolic and diastolic function, and clinical parameters.

## Methods

**Figure 1:**
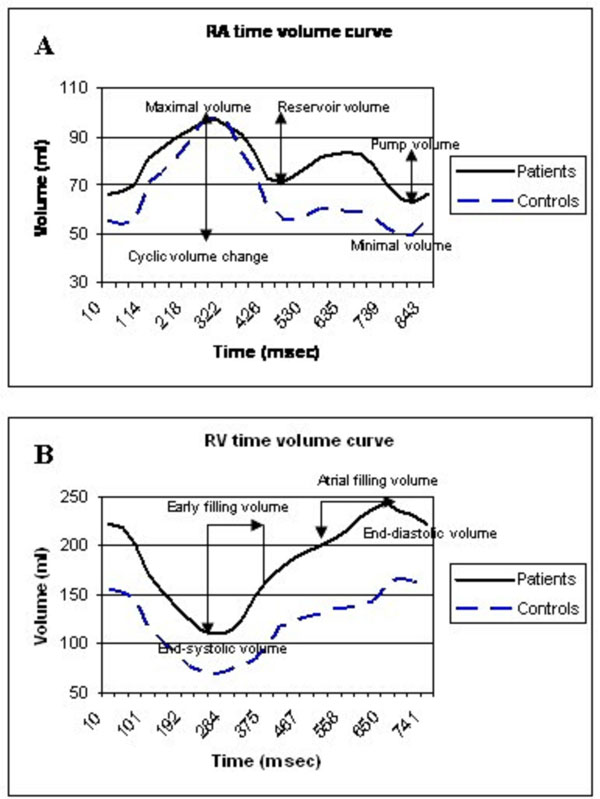
A) Right Atrial (RA) time-volume curve; B) Right Ventricular (RV) time-volume change curve.

51 patients (21±8 years) and 30 healthy controls (31±7 years) were included and underwent magnetic resonance imaging. Patients also underwent exercise testing, and biomarker assessment. Bi-atrial and biventricular size, systolic and diastolic function were assessed from time-volume curves (figure 1) and time-volume-change curves.

## Results

In patients, right atrial (RA) minimal volume (min.vol.), RA pump function, and RA late emptying fraction were increased (RA min.vol.: 34±8 ml/m^2^ (patients) vs. 28±8 ml/m^2^ (controls), p=0.001); RA reservoir function, RA early emptying fraction, and the RA early-to-late-emptying ratio (E/A ratio) were decreased (RA E/A ratio: 1.1±0.5 (patients) vs. 1.9±0.9 (controls), p<0.001). Patients had larger right ventricular (RV) volumes, lower biventricular ejection fraction and prolonged biventricular deceleration time (Dt) (RV Dt: 0.24±0.10 (patients) vs. 0.13±0.04 (controls), p<0.001). Left atrial (LA) maximal volume and LA early emptying fraction were decreased. Left ventricular (LV) E/A-filling ratio was increased (LV E/A-ratio: 4.2±3.7 (patients) vs. 2.7±1.3 (controls), p=0.008).

Patients with end-diastolic forward flow (EDFF) had larger RA and RV size, higher RA pump function (RA pump function: 27±8% (patients with EDFF) vs. 20±7% (patients without EDFF), p=0.003), higher N-terminal prohormone brain natriuretic peptide (NT-proBNP) levels, and higher ventilatory response to carbon dioxide production (VE/VCO_2_ slope: 32±4 (patients with EDFF) vs. 28±6 (patients without EDFF), p=0.014). Patients with abnormal RA emptying (reservoir function <30% and pump function >24%) had higher NT-proBNP levels (NT-proBNP: 16±10 pmol/l (abnormal RA emptying) vs. 7±7 pmol/l (normal RA emptying), p=0.002) and worse exercise capacity. RA min.vol. was positively associated with RV end-diastolic volume (r=0.35, p=0.013).

## Conclusions

In TOF patients with good clinical condition and moderate RV dilatation, abnormal bi-atrial function and abnormal biventricular diastolic function is common. Abnormal RA emptying was associated with impaired clinical outcome, as was the presence of EDFF. These parameters, together with RA enlargement, could serve as useful markers for clinically relevant RV diastolic dysfunction.

## Funding

This research was funded by the Netherlands Heart Foundation: grant 2006B095.

